# Characterization of HIV-1 CRF90_BF1 and putative novel CRFs_BF1 in Central West, North and Northeast Brazilian regions

**DOI:** 10.1371/journal.pone.0178578

**Published:** 2017-06-19

**Authors:** Mônica Nogueira da Guarda Reis, Gonzalo Bello, Monick Lindenmeyer Guimarães, Mariane Martins Araújo Stefani

**Affiliations:** 1 Laboratório de Imunologia da AIDS e da Hanseníase, Instituto de Patologia Tropical e Saúde Pública, UFG, Goiânia, Brazil; 2 Laboratório de AIDS e Imunologia Molecular, Instituto Oswaldo Cruz, FIOCRUZ, Rio de Janeiro, Brazil; University of Malaya, MALAYSIA

## Abstract

The Brazilian AIDS epidemic has been characterized by an increasing rate of BF1 recombinants and so far eight circulating recombinant forms/CRFs_BF1 have been described countrywide. In this study, *pol* sequences (protease/PR, reverse transcriptase/RT) of 87 BF1 mosaic isolates identified among 828 patients living in six Brazilian States from three geographic regions (Central West, North, Northeast) were analyzed. Phylogenetic and bootscan analyses were performed to investigate the evolutionary relationship and mosaic structure of BF1 isolates. Those analyses showed that 20.7% of mosaics (18 out of 87) were CRFs-like isolates, mostly represented by CRF28/CRF29_BF-like viruses (14 out of 18). We also identified five highly supported clusters that together comprise 42 out of 87 (48.3%) BF1 sequences, each cluster containing at least five sequences sharing a similar mosaic structure, suggesting possible new unidentified CRFs_BF1. The divergence time of these five potential new CRFs_BF1 clusters was estimated using a Bayesian approach and indicate that they probably originated between the middle 1980s and the middle 1990s. DNA was extracted from whole blood and four overlapping fragments were amplified by PCR providing full/near full length genomes (FLG/NFLG) and partial genomes. Eleven HIV-1 isolates from Cluster # 5 identified in epidemiologically unlinked individuals living in Central West and North regions provided FLG/NFLG/partial genome sequences with identical mosaic structure. These viruses differ from any known CRF_BF1 reported to date and were named CRF90_BF1 by the Los Alamos National Laboratory. This is the 9^th^ CRF_BF1 described in Brazil and the first one identified in Central West and North regions. Our results highlight the importance of continued molecular screening and surveillance studies, especially of full genome sequences to understand the evolutionary dynamics of the HIV-1 epidemic in a country of continental dimensions as Brazil.

## Introduction

Human Immunodeficiency Virus-1 (HIV-1) is a highly polymorphic and fast evolving pathogen [1]. Worldwide HIV-1 can be classified into groups (M, N, O and P), and the pandemic group M is classified in subtypes (A-D, F-H, J and K) and sub-subtypes (A1-A4, F1-F2) [2,3]. While mutation rates are similar to other RNA viruses, HIV-1 has a high recombinogenic capacity and intersubtype recombination events are frequent in coinfected or superinfected individuals from areas where two or multiple variants cocirculate [4]. Recombinant strains exhibiting identical mosaic patterns identified in at least three epidemiologically unlinked individuals have been classified as circulating recombinant forms (CRFs), while the ones displaying unique mosaic structures or only infecting individuals with epidemiological link are known as unique recombinant forms (URFs) [5,6]. Recombination has been recognized as a driving force in shaping the diversity of HIV-1 globally since the mid 90´s [7]. Currently, 88 CRFs have been assigned and 81 of them have been published with public data available at the Los Alamos HIV database [http://www.hiv.lanl.gov/content/sequence/HIV/CRFs/CRFs.html]. CRFs together with URFs are estimated to account for at least 20% of HIV-1 infections worldwide [8].

The Brazilian AIDS epidemic is characterized by the cocirculation of multiple HIV-1 subtypes. Subtype B predominates in most regions followed by subtypes F1, C, and recombinants among these subtypes [9–15]. The first Brazilian BF1 mosaics were identified in the early 90´s in the Southeast region which is considered the epicenter of the epidemic [16,17]. Among the 14 CRFs_BF1 described so far, eight originated in Brazil (CRF28_BF, CRF29_BF, CRF39_BF, CRF40_BF, CRF46_BF, CRF70_BF, CRF71_BF and CRF72_BF) [18–22]. The importance of BF1 recombinants in Brazil is further corroborated by the description of countless URFs in all country regions [23–26]. Previous studies from our research group in different study populations in Central West, North and Northeast Brazilian States showed variable prevalence of BF1 recombinants in the *pol* subgenomic fragment: (Goiás: 3.7–18.1%, Mato Grosso: 11.9%, Mato Grosso do Sul: 8.2–25.9%, Tocantins: 7.7%, Maranhão: 7.5%, Piauí: 4.5% [11,27–37].

In this study, previously produced *pol* sequences of BF1 mosaic isolates circulating in Central West, North and Northeast Brazil were reclassified into possible CRFs or URFs. Full/near full-length genome (FLG/NFLG) and partial genome sequences were obtained for the most representative potential CRF detected. These analyzes allowed the identification of the novel CRF90_BF1 that is circulating in Central West and North Brazil, away from the epicenter of the epidemic. Other putative novel CRFs_BF1 are also described. The median time of origin of these mosaics was also estimated. The detailed molecular characterization of recombinant forms circulating countrywide contributes to the mapping of HIV-1 diversity in Brazil.

## Material and methods

### Study population

Previous studies from our group recruited from 2003 to 2013 a total of 828 individuals infected with HIV-1 residing in six Brazilian States located in three geographic regions (Central West: Goiás/GO, Mato Grosso/MT, Mato Grosso do Sul/MS; North: Tocantins/TO; Northeast: Maranhão/MA, Piauí/PI) (**S1 Table**) [11,27–37]. These studies have identified a total of 87 (10.5%) BF1 recombinant isolates based on sequencing of *pol* subgenomic fragment covering the protease (PR) and partial reverse-transcriptase (RT) (positions 2253–3251 relative to HXB2 genome). The related research protocols were approved by the institutional Ethics Committee review boards (Goiás: protocols #073/05, #003/2008, #163/2010 at CEPMHA/HC/UFG, Mato Grosso: protocol #435/07 at Universidade Federal do Mato Grosso/UFMT, Mato Grosso do Sul: protocol #1143 at Universidade Federal do Mato Grosso do Sul/UFMS, Piauí; protocol #022/2011 at Universidade Estadual do Piauí/UESPI, Maranhão: protocol #16/2011 at Hospital de Doenças Tropicais Dr Natan Portela). All patients signed an informed consent form before blood collection for HIV-1 molecular studies.

### Amplification of HIV-1 PR/RT

RNA extraction, reverse transcription into complementary DNA (cDNA) and amplification by nested polymerase chain reaction (nested-PCR) of the PR/RT regions were previously described [11,27–37].

### Amplification of HIV-1 full length genomes

Genomic DNA was extracted from whole blood samples (QIAamp® DNA Blood Mini Kit/QIAGEN, Qiagen, Hilden, Germany). The complete HIV-1 genome was amplified by nested-PCR employing Platinum Taq DNA polymerase enzyme (Invitrogen, Carlsbad, CA) into four overlapping fragments using HIV-1 specific primers, as following: fragment 1- SCAOSD/LR51 external primers and SCANSD/DP11 internal primers (408–2594), fragment 2- DP10/SCCNAS external primers and DP16/SCCOAS internal primers (2253–4830); fragment 3- MMINT8/ED14 external primers and MMINT3/ED12 internal primers (4653–7811); fragment 4- ED5/SCDOAD external primers and JH44/LTR2 internal primers (6954–9625) (**S2 Table**) [38–40], all positions were relative to HXB2 genome. Isolates with all four fragments completely sequenced were considered full length genomes (FLG); isolates with three complete fragments were considered near full length genomes (NFLG), and isolates with one or two fragment sequences were referred as partial genomes.

### DNA sequencing

The amplified DNA fragments from the nested-PCR products were separated by gel electrophoresis, purified (kit QIAquick® PCR Purification Kit/QIAGEN, Qiagen, Hilden, Germany) and sequenced with the *Big Dye Terminator Sequencing Kit v*. *3*.*1* (Applied Biosystems, Foster City, CA) in an automated ABI Prism 3100 Genetic Analyzer (Applied Biosystems, USA). Chromatograms were analyzed and edited using the SeqMan software from the package DNASTAR Lasergene (MA, USA).

### Phylogenetic and recombination analyses

Sequences were aligned using Clustal X 2.0 implemented in BioEdit 7.2.0 program [41]. Reference sequences of HIV-1 group M subtypes (A-D, F–H, J and K) and CRF-BF1 sequences were obtained from the Los Alamos HIV database (http://hiv.lanl.gov/). Phylogenetic trees were generated using the neighbor-joining (NJ) method [42] under the Kimura two-parameter model [43] using MEGA 6.0 software [44]. Bootstrap values (BP, 1.000 replicates) above 70% were considered significant. Recombination analyses were performed in all viral isolates using bootscan implemented in Simplot v3.5.1 software with the following parameters: 200nt or 300nt window, 20nt increments, NJ method under Kimura’s two-parameter correction with 100 bootstrap replicates [45]. In this study the parameters used for bootscan analyses of recombinant viruses differed for smaller and larger fragments: for the analyses of pol fragments (998nt) a smaller sliding window of 200nt was used whereas for larger fragments of near full-genomes (>6670nt) a larger sliding window of 300nt was adopted. To better characterize the recombination breakpoints suggested in the previous analyses, the putative recombinants were subjected to informative site analyses as described elsewhere [39]. For this purpose, consensus sequences from Brazilian HIV-1 subtypes B and F were generated in the DAMBE program [46]. Fragments of sequences assigned to specific HIV-1 subtypes were finally confirmed by separate NJ phylogenetic analysis as described above.

Representative samples from the HIV-1 BF1 Brazilian clusters herein identified were submitted to a Basic Local Alignment Search Tool (BLAST) analysis in order to recover other Brazilian sequences with high similarity (>95%) and probably similar recombination profile. The BLAST analysis was done sequences using sequences obtained from the Los Alamos HIV database (http://hiv.lanl.gov/).

### Evolutionary analyses of BF1 recombinants

The time of the most recent common ancestor (T_MRCA_) of HIV-1 BF1 clades was estimated using a Bayesian Markov Chain Monte Carlo (MCMC) approach implemented in BEAST v1.8 [47,48] with BEAGLE to improve run-time [49]. Analyses were performed using the GTR+I+G nucleotide substitution model, a Bayesian Skyline coalescent tree prior [50] and a relaxed uncorrelated lognormal molecular clock model [51] with an informative uniform prior interval (1.0–3.0 x 10^−3^ nucleotide substitutions per site per year). One MCMC chain was run for 1x10^7^ generations. Convergence and uncertainty of parameter estimates were assessed by calculating the effective sample size (ESS) and the 95% highest probability density (HPD) values, respectively using Tracer v1.6 [52]. The maximum clade credibility (MCC) tree was summarized with TreeAnnotator v1.8 and visualized with FigTree v1.4.0.

### Data availability

All HIV-1 sequences generated in this study were deposited in the GenBank database (KY628215-KY628225).

## Results

### Phylogenetic and evolutionary analyses of BF1 *pol* recombinants

**Fig 1:**
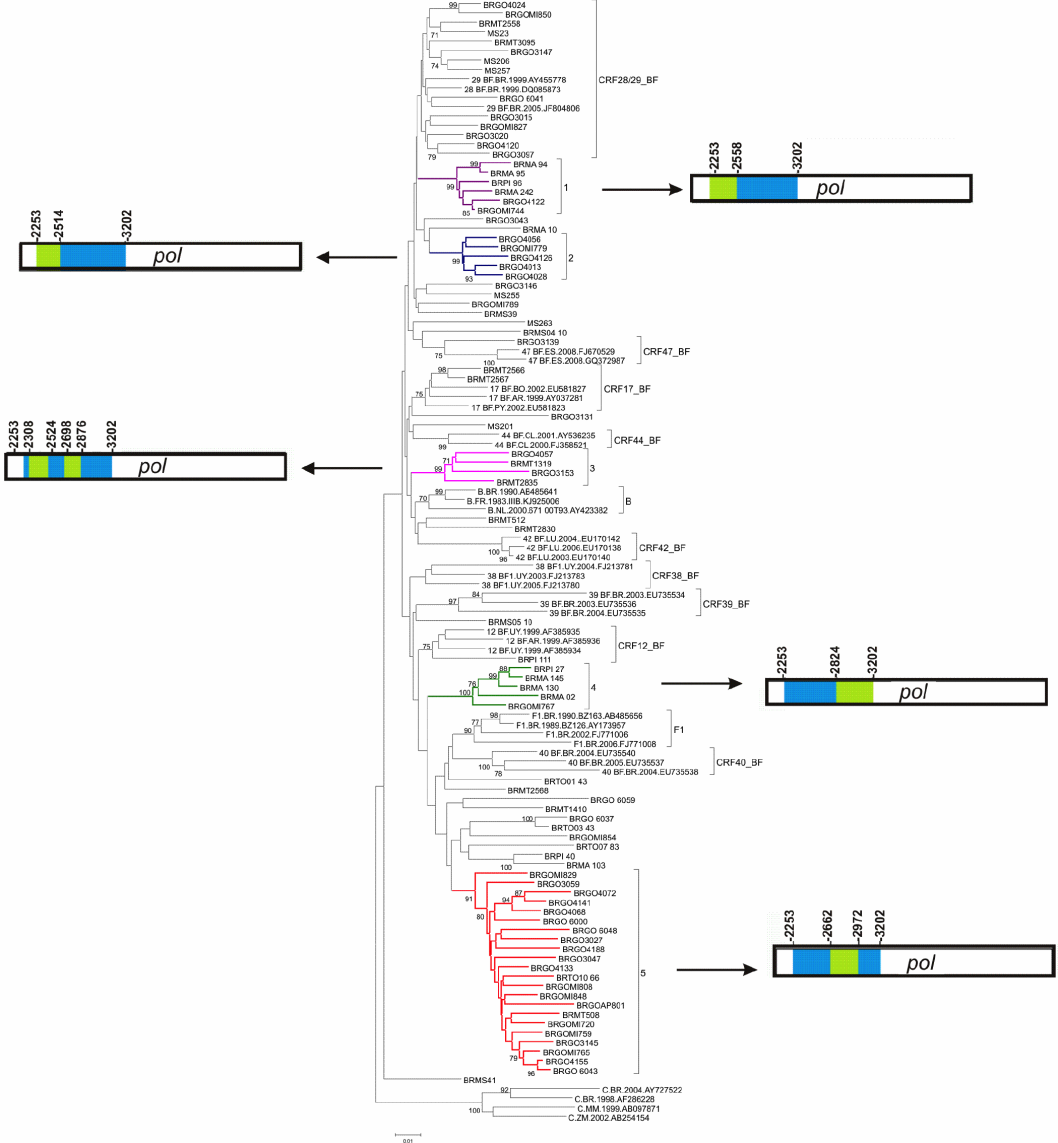
Phylogenetic analysis of 87 *pol* sequences of B/F1 HIV-1 isolates presenting five highly supported clusters and the mosaic pattern of recombination in each cluster (neighbor-joining method, Kimura 2-parameters evolutive model/1000 replicate *bootstrap* values). Bootscanning analyses of BF1 inter-subtype recombinant clusters (# 1–5) are represented. The five clusters identified in our study are indicated by different colors: Cluster # 1: purple, Cluster # 2: blue, Cluster # 3: pink, Cluster # 4: green and Cluster # 5: red. Bootscan analysis was performed in a 200nt sliding window advanced in 20nt step size increments (1.000 replicates). All CRF_BF depicting recombination breakpoints in *pol* region were included in the analysis. In the mosaic structure representations of BF1 isolates, the breakpoint positions according to HXB2 genome numeration are shown on the right and left sides of the clusters, blue stands for subtype B and green stands for subtype F.

Initial phylogenetic analyses of 87 HIV-1 isolates previously characterized as BF1 recombinants in the PR/RT region (**S1 Table**) classified 18 (21%) sequences as CRF_BF-like (14 CRF28/CRF29_BF-like, two CRF17_BF-like, one CRF12_BF-like and one CRF47_BF-like) and 27 (31%) sequences as URFs_BF (**Fig 1**). The remaining 42 (48%) sequences were distributed in five clusters comprising between five and 22 sequences, sharing the same mosaic structure and were classified as potential news CRFs_BF1 (**Fig 1**). Clusters # 1, 3 and 4 displayed high supports (BP ≥ 99%) at initial analysis. For Clusters # 2 and 5, however, high supports were obtained only after exclusion of the URFs_BF MS251, BRGO3127 and BRGO4162 sequences (**Fig 1**). Cluster # 1 had six sequences, from three different States (two from Goiás, three from Maranhão and one from Piauí). Cluster # 2 had five sequences, all from Goiás State. Cluster # 3 comprised four sequences from two States (two from Mato Grosso and two from Goiás). Cluster # 4 had five sequences from three States (one from Goiás, three from Maranhão and one from Piauí). Cluster # 5 contained 22 sequences from three States (20 from Goiás, one from Mato Grosso and one from Tocantins).

**Fig 2:**
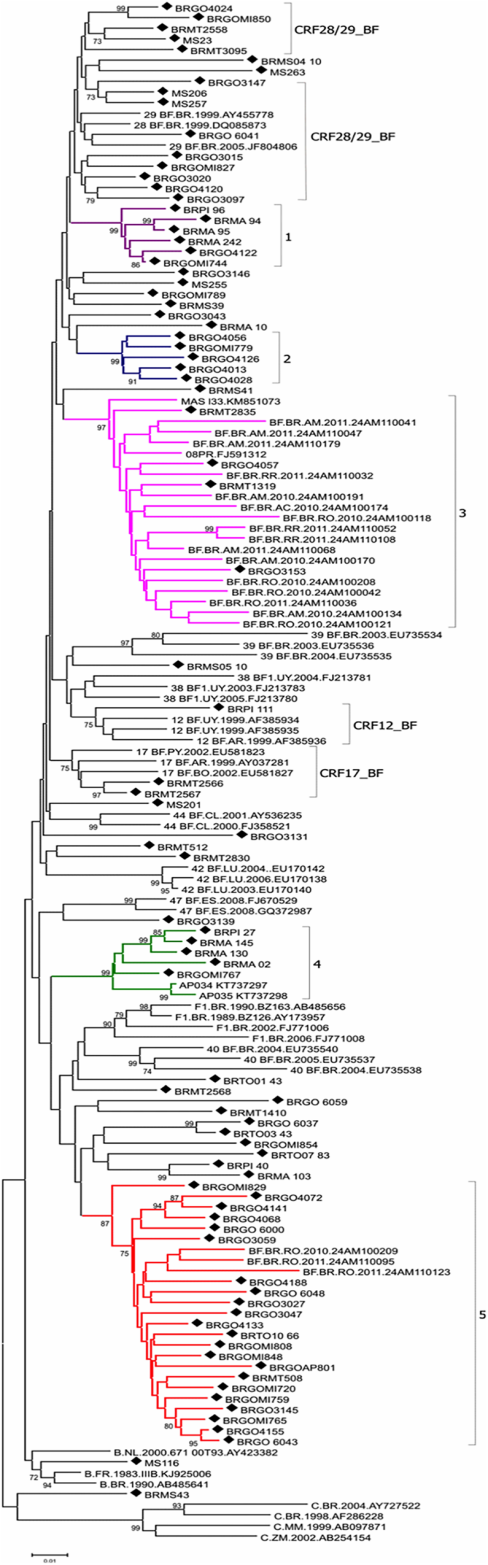
Phylogenetic tree of study BF1 isolates from Central West, North, Northeast and South Brazil and BF1 sequences from GenBank sharing over 95% similarity with study isolates. Trees were constructed using MEGA software, 6.0 version under neighbor-joining and Kimura 2 parameters methods (Bootstrap value over 70%). The sequences described in our study are distinguished from the sequences retrieved from the GenBank by a diamond signal. The five clusters identified in our study are indicated by different colors: Cluster # 1: purple, Cluster # 2: blue, Cluster # 3: pink, Cluster # 4: green and Cluster # 5: red.

A Blast search analysis was performed to identify sequences similar to the five potential new CRF_BF1 Brazilian clusters. The recovered sequences were included in the phylogenetic and recombinant analysis, bootstrap values higher than 87% and similar mosaic profiles compared to those previously classified in Clusters # 3, 4 and 5 was verified (**Fig 2**). Eighteen sequences branching within Custer # 3 were recovered from patients recruited in four States from the North region (seven from Amazonas, five from Rondônia, three from Roraima and one from Acre) along with two sequences from the South region (Paraná) (**Fig 2**). Two sequences from the North region (Amapá) classified in Cluster # 4 and three sequences classified in Cluster # 5 were recovered from patients from the North region (Rondônia) (**Fig 2**).

**Fig 3:**
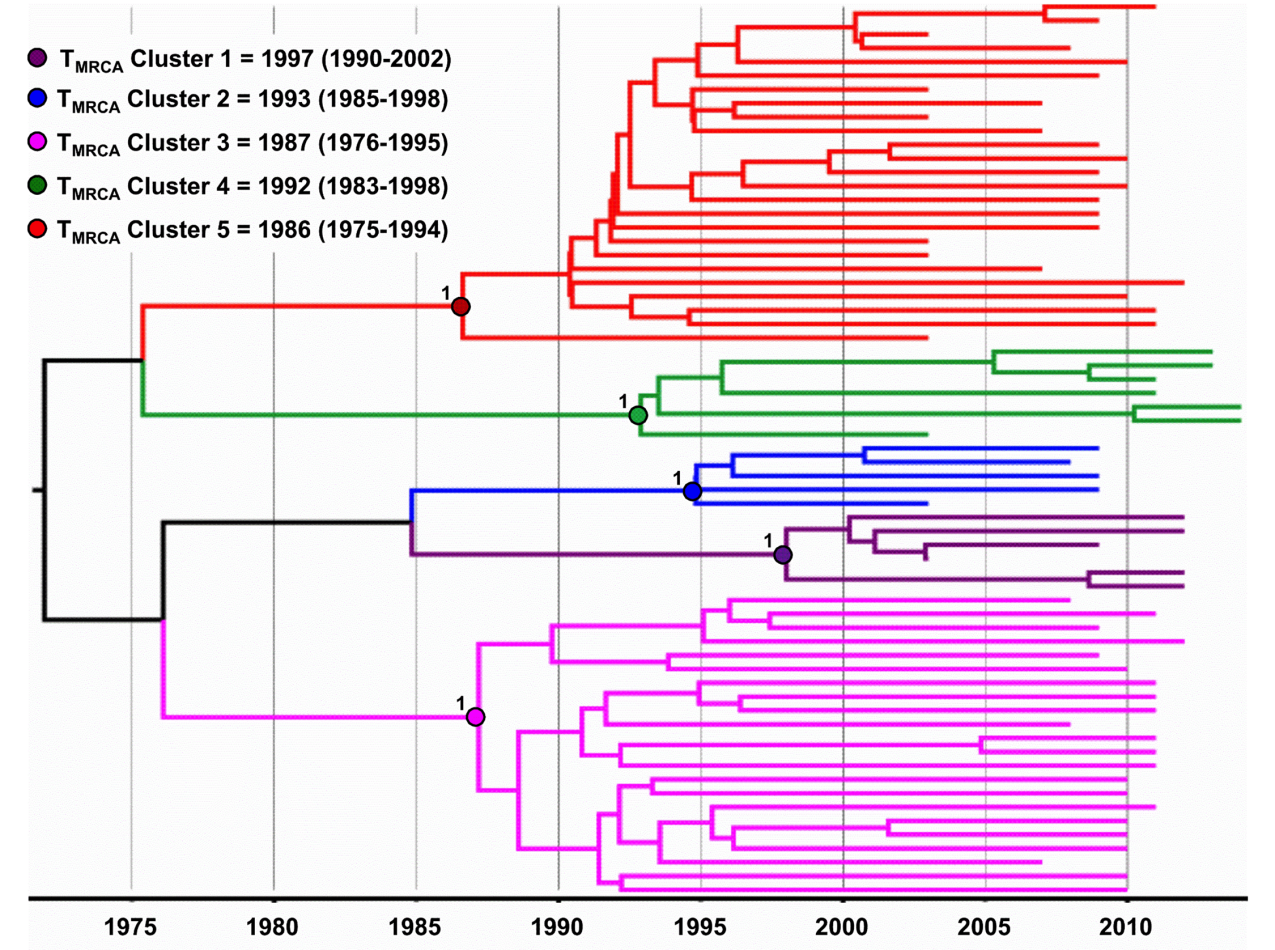
Time-scaled Bayesian MCMC tree of 65 *pol* sequences of BF1 HIV-1 isolates that grouped into five clusters from Central West, North, Norhteast and South Brazil. The circles indicate the positions of the MRCA of each BF1 cluster. Branch lengths are depicted in units of time (years). The tree was automatically rooted under the assumption of a relaxed molecular clock. The five clusters identified in our study are indicated by different colors: Cluster # 1: purple, Cluster # 2: blue, Cluster # 3: pink, Cluster # 4: green and Cluster # 5: red.

The Bayesian MCC tree displayed the same topology of the NJ tree, thus confirming the five BF1 phylogenetic clusters initially described (**Fig 3**). According to this analysis, the median T_MRCA_ of the five potential new Brazilian CRFs_BF identified was estimated between the middle 1980s and the middle 1990s (**Fig 3**).

### Analysis of FLG, NFLG and partial genomes

**Fig 4:**
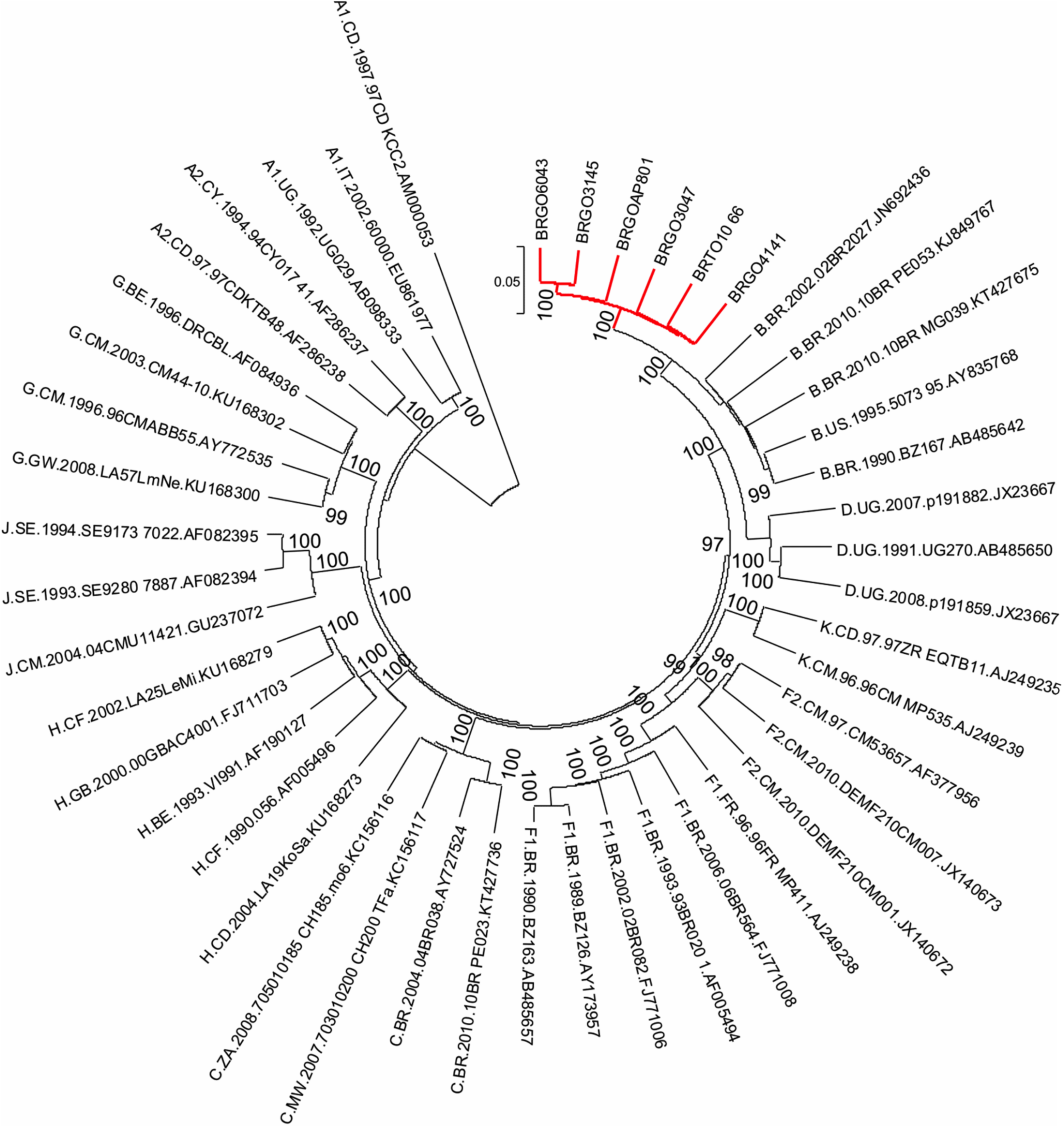
Phylogenetic analyses on the full length/near-full-length genome sequences of HIV-1 BF1 isolates from cluster # 5 belonging to a new CRF90_BF1 identified in patients from Goiás and Tocantins States in the Central West and North Brazilian regions. The HIV group M reference sequences of subtypes were obtained from the Los Alamos database. The scale bar represents 0.02 nucleotide substitutions per site. Phylogenic analyses were constructed with Mega software version 6.0.

**Fig 5:**
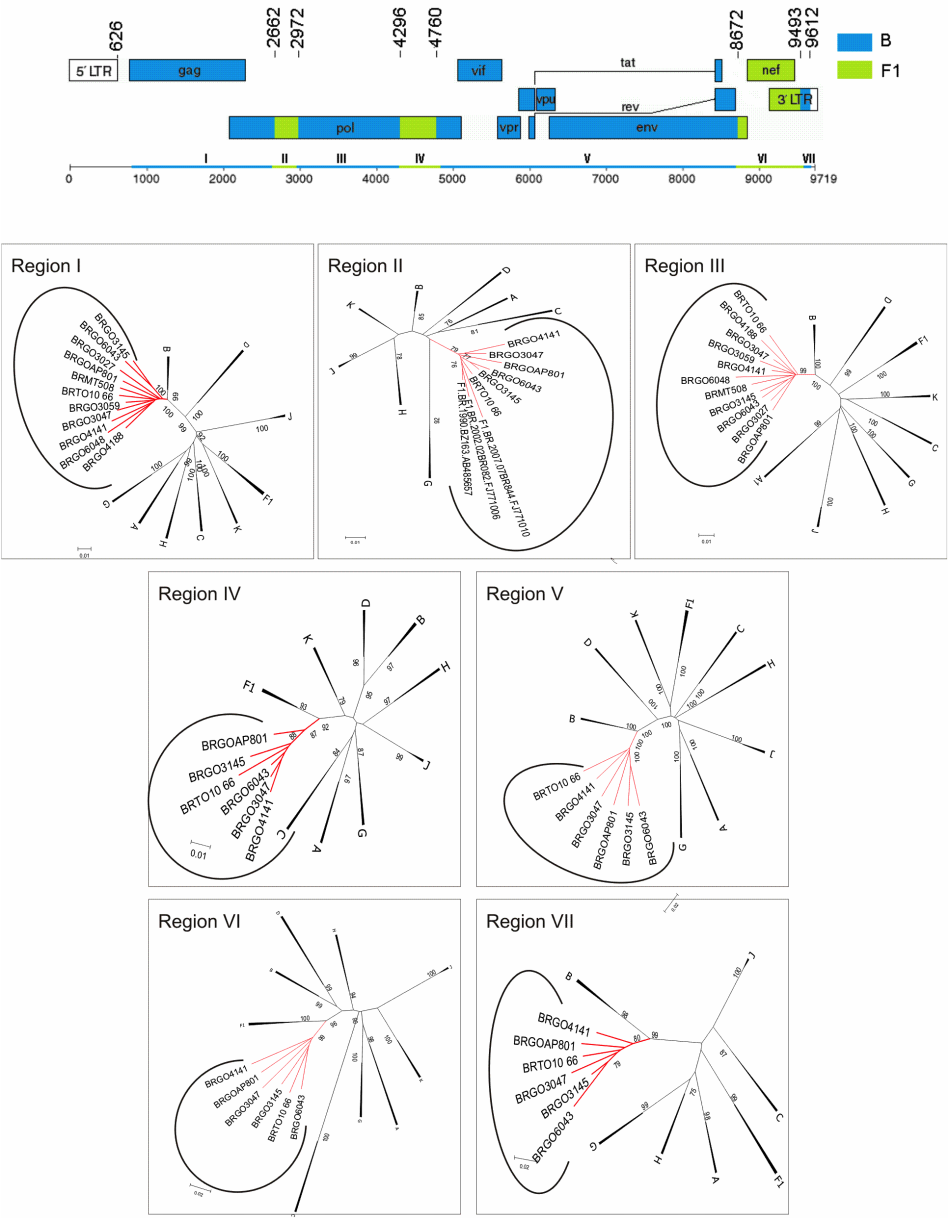
Mosaic structure of the new CRF composed by subtypes B and F1. **Breakpoint positions according to HXB2 genome numbering system are indicated**. The phylogenetic trees for each of the seven mosaic segments (I-VII) were constructed with Mega software v6.0 and the trees were midpoint rooted. The stability of each node was confirmed by bootstrapping with 1.000 replicates and only significant bootstrap values >70% are shown at the corresponding nodes. The genetic distance corresponding to the length of the branches is shown by the line at the bottom. The red color represents the CRF90_BF1 identified in this study.

Phylogenetic (**Fig 4**) and bootscan analyses of six full length genomes (BRGOAP801, BRGO6043, BRTO10_66, BRGO4141, BRGO3145 and BRGO3047) obtained from isolates classified in Cluster # 5 allowed the description of a new recombinant lineage designated CRF90_BF1 by the Los Alamos HIV Sequence Database (Los Alamos National Laboratory) according to the standardized nomenclature [2]. We also obtained one NFLG and four partial genomes for isolates from this Cluster that share the same mosaic structure (**Fig 5**). The mosaic structures inferred from the analyses of these FLG, NFLG and partial genomes showed a genome predominantly of subtype B, which can be divided into seven subregions alternating subtypes B and F1. These seven subregions were named I (626–2.661), II (2.662–2.971), III (2.972–4.295), IV (4.296–4.759), V (4.760–8.671), VI (8.672–9.492) and VII (9.493–9.612) all positions relative to HXB2 genome. Subregion NJ analyses also confirmed the putative parental HIV-1 subtype (**Fig 5**). Fully coincident intersubtype breakpoint locations at I-III sub regions were also observed in the NFLG of BRGO4188 isolate and in the partial genome sequences of BRMT508, BRGO3027, BRGO3059 and BRGO6048 isolates (**Fig 5 and Table 1**).

**Table 1:** Demographic characteristics of study subjects infected by HIV-1 CRF90_BF1.

Sample ID	ARV status	Gender	Age	Risk Group	HIV Diagnosis (Year)	Sample Collection (Year)	HXB2 location (nt)	Accession Number
BRGOAP801	HAART	M	59	Heterosexual	2007	2010	407–9626	KY628223
BRGO6043	Naïve	F	30	Heterosexual	2011	2011	407–9626	KY628221
BRTO10_66	Naïve	F	27	Heterosexual	2009	2009	407–9616	KY628225
BRGO4141	Prophylaxis	F	24	Heterosexual	2010	2010	408–9615	KY628219
BRGO3145	HAART	F	35	Heterosexual	2004	2007	407–9612	KY628218
BRGO3047	HAART	M	47	NA	2002	2007	474–9589	KY628216
BRGO4188	Prophylaxis	F	27	Heterosexual	2006	2010	407–7080*	KY628220
BRGO3027	Naïve	M	27	IDU	2002	2007	453–5924*	KY628215
BRMT508	Naïve	M	41	Heterosexual	2009	2009	414–5919*	KY628224
BRGO3059	Naïve	M	51	Heterosexual	2007	2007	407–4778*	KY628217
BRGO6048	Naïve	F	30	Heterosexual	2009	2012	454–4122*	KY628222

M: Male; F: Female; Naïve: antiretroviral naïve patients; HAART: Patients under highly active antiretroviral therapy; Prophylaxis: mother-to-child-transmission antiretroviral prophylaxis (MTCT ARV prophylaxis); IDU: intravenous drug user; N.A.: not available

* isolates with partial genome sequences; Isolates are listed according to the size of sequenced fragments. nt: nucleotide position

The epidemiological features of the 11 patients presenting the newly described CRF90_BF1 lineage included six females (four of them pregnant) and five males (two of them prisoners) (**Table 1**). The prevailing risk category was heterosexual sex reported by nine patients while one prisoner patient reported intravenous drug use. Six patients were ARV naïve and five had been exposed to ARV drugs either as highly active antiretroviral therapy (HAART) or temporary mother-to-child-transmission (MTCT) prophylaxis. Most patients were from the Central West region (Goiás State: isolates BRGO3027, BRGO3047, BRGO3145, BRGO3059, BRGO4188, BRGO4141, BRGO6048, BRGOAP801 and BRGO6043; Mato Grosso State: isolate BRMT508) and one patient lived in the North region (Tocantins State: isolate BRTO10_66).

## Discussion

In this study, we report the characterization of a novel HIV-1 CRF_BF1, named CRF90_BF1 based on six FLG, one NFLG and four partial genome sequences. These isolates shared identical mosaic structures and were identified in individuals without any epidemiological link that live in two distinct geographic regions in Brazil (Central West and North) located around 800–900 km apart. These criteria fulfill the requirements to define a new CRF, which is circulating in distant interior urban areas in Brazil. This novel CRF is the 9^th^ CRF involving subtypes B and F1 described in Brazil and the 14^th^ reported in South America. The estimated frequency of the CRF90_BF1 in our sample set was 1.3% (11/828), with predominant detection in the Central West region. However, the actual prevalence of this new CRF in these geographic regions cannot be accurately estimated since there is limited molecular data on HIV-1 isolates especially from the States of Mato Grosso, Mato Grosso do Sul and Tocantins.

The CRF12_BF, the first CRF identified in the Americas was described in 2001 in patients from Argentina and Uruguay and its origin was estimated around the early 80s [53,54], while BF1 recombinants were first reported in Brazil in the early 90’s [16,17]. Patients harboring the CRF90_BF1 were diagnosed between 2002 and 2011. The median estimated T_MRCA_ of the CRF90_BF1 and of other putative CRF_BF1 clusters identified in our study is not recent and ranges from middle 80’s to middle 90’s, similar to that previously estimated for Brazilian CRF28_BF and CRF29_BF [55]. These estimates indicate that CRFs_BF1 have been probably circulating in Brazil for three to four decades.

**Fig 6:**
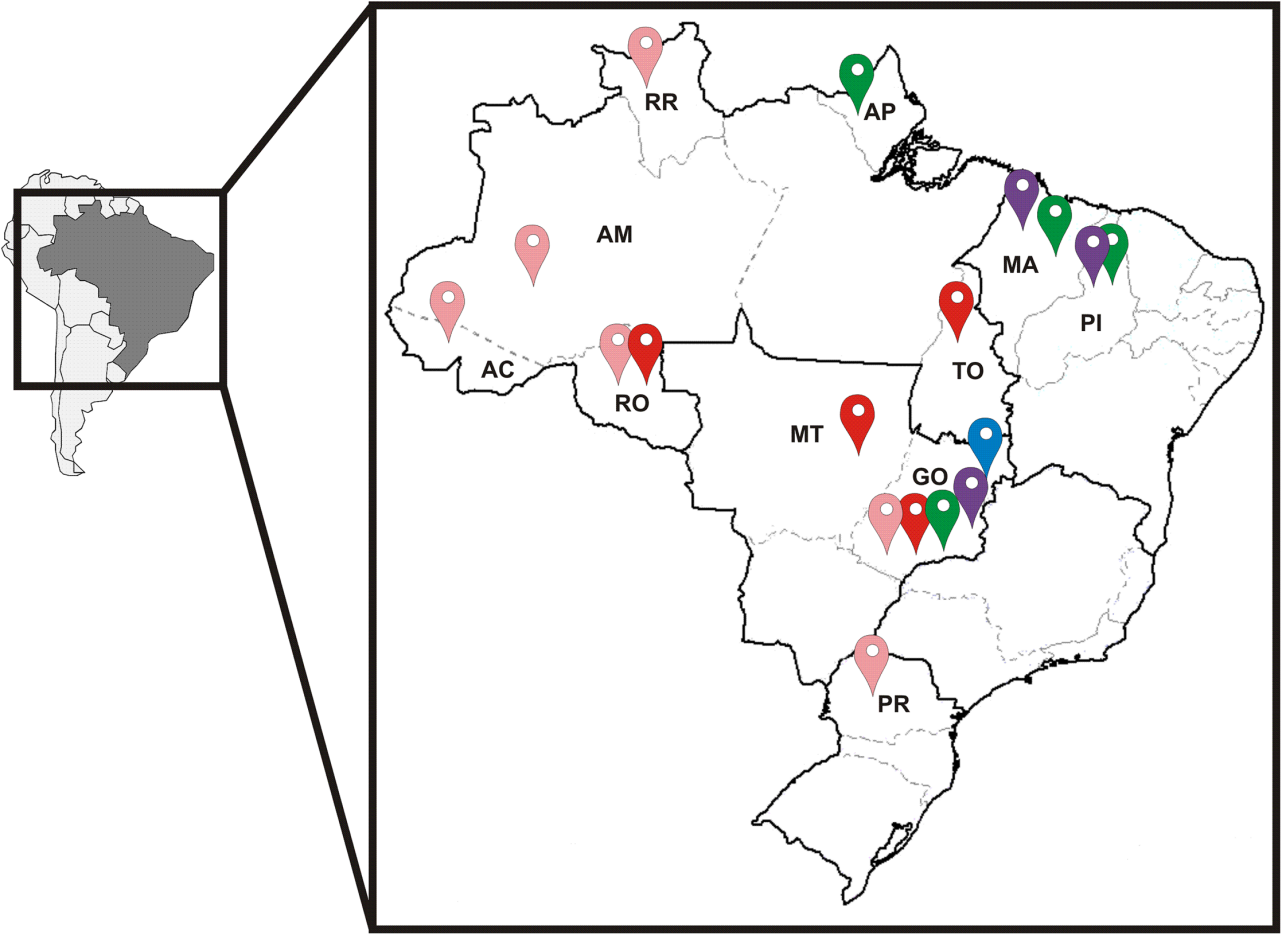
South America map highlighting Brazil. **Color marks indicate the possible geographical area of circulation of BF1 isolates identified in Central West, North, Northeast and South Brazil and BF1 sequences from GenBank sharing over 95% similarity with study isolates from Clusters # 1–5.** In the Brazilian map, each colored mark represents the geographic area of potential circulation of: Cluster # 1-purple, Cluster # 2-blue, Cluster # 3-pink, Cluster # 4-green, Cluster # 5-red.

Besides its early generation, we have evidences, as shown by blast search analyses, that the CRF90_BF1 and also the other putative CRFs_BF1 clades identified here have a wide geographic circulation (**Fig 6**). The CRF90_BF1 that we identified in Central West (Goiás and Mato Grosso) and North Brazil (Tocantins) is probably also circulating in Rondônia, another State in the North region which borders Bolivia in the South/East. HIV-1 BF1 isolates with the same recombination pattern of isolates from Cluster # 3 detected in Central West were also identified in several North Brazilian States (Amazonas, Rondônia, Roraima and Acre), and in the South State of Paraná. Isolates with similar recombination profile of isolates from Cluster # 4 were also identified in the North region (Amapá State) besides the Central West (Goiás) and Northeast Brazil (Maranhão and Piauí). These results suggest the existence of novel CRFs_BF1 circulating in Brazil.

CRF28_BF and CRF29_BF described in the Southeast in 2006 (Santos, São Paulo State) represent the first Brazilian CRFs, and their origin date to 1988–1989 [18,55]. Studies have shown a low prevalence of CRF28_BF and CRF29_BF [14,56], outside São Paulo except in Salvador, Bahia State, Northeast where prevalence ranged from 10%-21% [57,58]. Among all BF1 isolates identified in our study we have found a moderate rate of CRF28/CRF29_BF-like isolates (16.1%, 14 out of 87) and an overall rate of 1.7% (14 out of 828) which represent one of the highest frequencies of these CRFs identified outside São Paulo State.

Despite the predominance of subtype B in most geographic Brazilian regions, except in the South where subtype C prevails, studies have shown that the prevalence of non-B subtypes, particularly URFs_BF1 and URFs_BC has increased in the last decade [15,25,40,59,60]. Our studies have shown a significant percentage of recombinant BF1 forms (3.7–25.9%) in the Central West, North and Northeast Brazilian regions [11,27–37]. The most recently described Brazilian CRFs_BF1 (CRF70_BF1 and CRF71_BF1) were identified among blood donors from Pernambuco State, Northeast region [22]. The CRF72_BF1 was identified among blood donors from five public blood banks in Minas Gerais State, Southeast region [21]. These recent data point out the increasing generation and spread of CRFs, especially involving subtypes B and F1 which play an important role in the Brazilian AIDS epidemic. However, the number of complete genome sequences available is still limited, especially sequences from areas away from the epicenter, as our study areas (Central West, North and Northeast) suggesting that the actual proportion of HIV-1 recombinant forms in the Brazilian pandemic is probably underestimated.

## Conclusions

In summary, we identified the novel CRF90_BF1 among heterosexual patients living in two geographic regions in Brazil, away from the epicenter of the epidemic. This is the 9^th^ CRF_BF1 described in Brazil indicating that continued molecular screening and surveillance are necessary to fully understand the evolutionary dynamics of the HIV-1 epidemic in such a country of continental dimensions. Our results also underscore the importance of full-length genome sequencing of HIV-1 isolates obtained from patients infected by different transmission routes and in different country regions to fully understand the diversity and complexity of the HIV-1 epidemic in Brazil.

## Supporting information

S1 Table Prevalence of BF1 recombinants identified in previous studies among patients from six Brazilian States: Goiás/ GO, Mato Grosso/ MT, Mato Grosso do Sul/MS, Tocantins/TO, Piauí/PI and Maranhão/MA. Pregnant: women infected with HIV-1 attending a regional antenatal care; Naïve: antiretroviral naïve patients; HAART: Patients under highly active antiretroviral therapy. * Ref 29: the study group (n = 27) comprises prisoner patients recruited in Goiania/GO (n = 7) and in Campo Grande (n = 20).
(DOCX)

S2 Table List of HIV-1 primers used in the present study for full length genome amplification. *Some primers had their original sequence modified based on the alignment of subtypes B, C and F1 HIV sequence compendium (2005) from HIV Los Alamos Database.
(DOCX)

## References

[1] Robertson DL, Hahn BH, Sharp PM. Recombination in AIDS viruses. J Mol Evol. 1995; 40(3):249–59. 7723052 10.1007/BF00163230

[2] Robertson DL, Anderson JP, Bradac JA, Carr JK, Foley B, Funkhouser RK, et al HIV-1 nomenclature proposal. Science. 2000; 288(5463):55–56 10766634 10.1126/science.288.5463.55d

[3] Tebit DM and Arts EJ. Tracking a century of global expansion and evolution of HIV to drive understanding and to combat disease. Lancet Infect Dis. 2011; 11(1):45–56. doi: 10.1016/S1473-3099(10)70186-9 21126914 10.1016/S1473-3099(10)70186-9

[4] Thomson MM, Nájera R. Molecular epidemiology of HIV-1 variants in the global AIDS pandemic: an update. AIDS Rev. 2005;7(4):210–24. 16425961

[5] Thomson MM, Pérez-Alvarez L, Nájera R. Molecular epidemiology of HIV-1 genetic forms and its significance for vaccine development and therapy. Lancet Infect Dis. 2002; 2(8):461–471. Review. 12150845 10.1016/s1473-3099(02)00343-2

[6] Nájera R, Delgado E, Pérez-Alvarez L, Thomson MM. Genetic recombination and its role in the development of the HIV-1 pandemic. AIDS. 2002; Suppl 4:S3–16. 10.1097/00002030-200216004-00002 12698994

[7] Gao F, Robertson D L, Morrison S G, Hui H, Craig S, Decker J, et al The heterosexual human immunodeficiency virus type 1 epidemic in Thailand is caused by an intersubtype (A/E) recombinant of African origin. J Virol. 1996; 70(10):7013–7029. 8794346 10.1128/jvi.70.10.7013-7029.1996 PMC190752

[8] Hemelaar J, Gouws E, Ghys PD, Osmanov S. WHO-UNAIDS Network for HIV Isolation and Characterization 2011. Global trends in molecular epidemiology of HIV-1 during 2000–2007. AIDS. 2011; 25(5):679–689. doi: 10.1097/QAD.0b013e328342ff93 21297424 10.1097/QAD.0b013e328342ff93 PMC3755761

[9] Brindeiro RM, Diaz RS, Sabino EC, Morgado MG, Pires IL, Brigido L, et al Brazilian Network for HIV Drug Resistance Surveillance (HIV-BResNet): a survey of chronically infected individuals. AIDS. 2003; 17(7):1063–9. doi: 10.1097/01.aids.0000060345.12269.d7 12700457 10.1097/00002030-200305020-00016

[10] Inocencio LA, Pereira AA, Sucupira MC, Fernandez JC, Jorge CP, Souza DF, et al Brazilian Network for HIV Drug Resistance Surveillance: a survey of individuals recently diagnosed with HIV. J Int AIDS Soc. 2009; 18;12:20. 10.1186/1758-2652-12-20 PMC2759910 19765271

[11] Cardoso LP, Queiroz BB, Stefani MM. HIV-1 pol phylogenetic diversity and antiretroviral resistance mutations in treatment naïve patients from Central West Brazil. J Clin Virol. 2009; 46(2):134–139. doi: 10.1016/j.jcv.2009.07.009 19682948 10.1016/j.jcv.2009.07.009

[12] Alencar CS, Sabino EC, Carvalho SM, Leao SC, Carneiro-Proietti AB, Capuani L, et al HIV genotypes and primary drug resistance among HIV-seropositive blood donors in Brazil: role of infected blood donors as sentinel populations for molecular surveillance of HIV. J Acquir Immune Defic Syndr. 2013;1;63(3):387–92. doi: 10.1097/QAI.0b013e31828ff979 23507660 10.1097/QAI.0b013e31828ff979 PMC4136640

[13] Silveira J, Santos AF, Martínez AM, Góes LR, Mendoza-Sassi R, Muniz CP, et al Heterosexual transmission of human immunodeficiency virus type 1 subtype C in southern Brazil. J Clin Virol. 2012; 54(1):36–41. doi: 10.1016/j.jcv.2012.01.017 22326760 10.1016/j.jcv.2012.01.017

[14] Guimarães ML, Marques BC, Bertoni N, Teixeira SL, Morgado MG, Bastos FI, et al Assessing the HIV-1 Epidemic in Brazilian Drug Users: Molecular Epidemiology Approach. PLoS One. 2015;10(11):e0141372 doi: 10.1371/journal.pone.0141372 26536040 10.1371/journal.pone.0141372 PMC4633026

[15] da Costa CM, Costa de Oliveira CM, Chehuan de Melo YF, Delatorre E, Bello G, Couto-Fernandez JC. High HIV-1 Genetic Diversity in Patients from Northern Brazil. AIDS Res Hum Retroviruses. 2016; 32(9):918–22. doi: 10.1089/AID.2016.0044 27091699 10.1089/AID.2016.0044

[16] Sabino EC, Shpaer EG, Morgado MG, Korber BT, Diaz RS, Bongertz V, et al Identification of human immunodeficiency virus type 1 envelope genes recombinant between subtypes B and F in two epidemiologically linked individuals from Brazil.J Virol. 1994; 68(10):6340–6. 8083973 10.1128/jvi.68.10.6340-6346.1994 PMC237055

[17] Ramos A, Tanuri A, Schechter M, Rayfield MA, Hu DJ, Cabral MC, et al Dualand recombinant infections: an integral part of the HIV-1 epidemic in Brazil. Emerg Infect Dis. 1999; 5(1):65–74. doi: 10.3201/eid0501.990108 10081673 10.3201/eid0501.990108 PMC2627691

[18] De Sá Filho DJ, Sucupira MC, Caseiro MM, Sabino EC, Diaz RS, Janini LM. Identification of two HIV type 1 circulating recombinant forms in Brazil. AIDS Res Hum Retroviruses. 2006; 22(1):1–13. doi: 10.1089/aid.2006.22.1 16438639 10.1089/aid.2006.22.1

[19] Guimarães ML, Eyer-Silva WA, Couto-Fernandez JC, Morgado MG. Identification of two new CRF_BF in Rio de Janeiro State, Brazil. AIDS. 2008; 22(3):433–435. doi: 10.1097/QAD.0b013e3282f47ad0 18195572 10.1097/QAD.0b013e3282f47ad0

[20] Sanabani SS, Pastena ER, Neto WK, Martinez VP, Sabino EC. Characterization and frequency of a newly identified HIV-1 BF1 intersubtype circulating recombinant form in São Paulo, Brazil. Virol J. 2010; 16;7:74. 10.1186/1743-422X-7-74 PMC2859377 20398371

[21] Pessôa R, Carneiro Proietti AB, Busch MP, Sanabani SS. Identification of a Novel HIV-1 Circulating Recombinant Form (CRF72_BF1) in Deep Sequencing Data from Blood Donors in Southeastern Brazil. Genome Announc. 2014a; 12;2(3). 10.1128/genomeA.00386-14 PMC4056286 24926043

[22] Pessôa R, Watanabe JT, Calabria P, Felix AC, Loureiro P, Sabino EC, et al Deep sequencing of HIV-1 near full-length proviral genomes identifies high rates of BF1 recombinants including two novel circulating recombinant forms (CRF) 70_BF1 and a disseminating 71_BF1 among blood donors in Pernambuco, Brazil. PLoS One. 2014b; 17;9(11):e112674. 25401747 10.1371/journal.pone.0112674 PMC4234413

[23] Guimarães ML, Couto-Fernandez JC, Eyer-Silva Wde A, Teixeira SL, Chequer-Fernandez SL, Morgado MG. Analysis of HIV-1 BF pr/rt recombinant strains from Rio de Janeiro/Brazil reveals multiple unrelated mosaic structures. Infect Genet Evol. 2010; 10(7):1094–100. doi: 10.1016/j.meegid.2010.07.001 20621204 10.1016/j.meegid.2010.07.001

[24] Sanabani SS, Pastena ÉR, da Costa AC, Martinez VP, Kleine-Neto W, de Oliveira AC, et al Characterization of partial and near full length genomes of HIV-1 strains sampled from recently infected individuals in São Paulo, Brazil. PLoS One. 2011; 6(10):e25869 doi: 10.1371/journal.pone.0025869 22022460 10.1371/journal.pone.0025869 PMC3193532

[25] Sanabani SS, Pessôa R, Soares de Oliveira AC, Martinez VP, Giret MT, de Menezes Succi RC, et al Variability of HIV-1 genomes among children and adolescents from São Paulo, Brazil. PLoS One. 2013; 8(5):e62552 doi: 10.1371/journal.pone.0062552 23667488 10.1371/journal.pone.0062552 PMC3646872

[26] Pessôa R, Loureiro P, Esther Lopes M, Carneiro-Proietti AB, Sabino EC, Busch MP, et al Ultra-Deep Sequencing of HIV-1 near Full-Length and Partial Proviral Genomes Reveals High Genetic Diversity among Brazilian Blood Donors. PLoS One. 2016; 11(3):e0152499 doi: 10.1371/journal.pone.0152499 27031505 10.1371/journal.pone.0152499 PMC4816342

[27] Cardoso LPV, Stefani MMA. High level of multidrug resistance mutations in HIV type 1 pol gene and resistance-associated mutations to enfuvirtide (T-20) among antiretroviral-experienced patients from central Brazil. AIDS Res Hum Retroviruses. 2009; 25:943–950. doi: 10.1089/aid.2009.0060 19792869 10.1089/aid.2009.0060

[28] Cardoso LPV, Pereira GAS, Viegas AA, Schmaltz LEPR, Stefani MMA. HIV-1 primary and secondary antiretroviral drug resistance and genetic diversity among pregnant women from Central Brazil. Journal of Medical Virology. 2010; 82:351–357. doi: 10.1002/jmv.21722 20087934 10.1002/jmv.21722

[29] Cardoso LP, da Silveira AA, Francisco RB, da Guarda Reis MN, Stefani MM. Molecular characteristics of HIV type 1 infection among prisoners from Central Western Brazil. AIDS Res Hum Retroviruses. 2011; 27(12):1349–1353. doi: 10.1089/aid.2011.0153 21732793 10.1089/aid.2011.0153 PMC3227242

[30] Ferreira AS, Cardoso LP, Stefani MM. Moderate prevalence of transmitted drug resistance and high HIV-1 genetic diversity in patients from Mato Grosso State, Central Western Brazil. J Med Virol. 2011; 83(8):1301–1307. doi: 10.1002/jmv.22128 21678433 10.1002/jmv.22128

[31] Carvalho BC, Cardoso LP, Damasceno S, Stefani MM. Moderate prevalence of transmitted drug resistance and interiorization of HIV type 1 subtype C in the inland North State of Tocantins, Brazil. AIDS Res Hum Retroviruses. 2011; 27(10):1081–1087. doi: 10.1089/AID.2010.0334 21417758 10.1089/AID.2010.0334

[32] Silveira AA, Cardoso LP, Francisco RB, de Araújo Stefani MM. HIV type 1 molecular epidemiology in pol and gp41 genes among naïve patients from Mato Grosso do Sul State, central western Brazil. AIDS Res Hum Retroviruses. 2012; 28(3):304–307. doi: 10.1089/aid.2011.0128 21790471 10.1089/aid.2011.0128 PMC3292747

[33] Alcântara KC, Lins JB, Albuquerque M, Aires LM, Cardoso LP, Minuzzi AL, et al HIV-1 mother-to-child transmission and drug resistance among Brazilian pregnant with high Access to diagnosis and prophylactic measures. Journal of Clinical Virology. 2012; 54:15–20. doi: 10.1016/j.jcv.2012.01.011 22317908 10.1016/j.jcv.2012.01.011

[34] da Costa ZB, de Lima YA, Martelli CM, Stefani MM. Transmitted HIV resistance among pregnant young women infected with HIV-1 in Brazil. AIDS Patient Care STDS. 2013; 27(8):439–41. doi: 10.1089/apc.2012.0448 23968204 10.1089/apc.2012.0448 PMC3739955

[35] Moura ME, Reis MN, Lima YA, Eulálio KD, Cardoso LP, Stefani MM. Low rate of transmitted drug resistance may indicate low access to antiretroviral treatment in Maranhão State, northeast Brazil. AIDS Res Hum Retroviruses. 2015a; 31(2):250–4. 25411830 10.1089/aid.2014.0261 PMC4313400

[36] Moura ME, da Guarda Reis MN, Lima YA, Eulálio KD, Cardoso LP, Stefani MM. HIV-1 transmitted drug resistance and genetic diversity among patients from Piauí State, Northeast Brazil. J Med Virol. 2015b; 87(5):798–806. 25649362 10.1002/jmv.24087

[37] Lima YA, Cardoso LP, Reis MN, Stefani MM. Incident and long term HIV-1 infection among pregnant women in Brazil: Transmitted drug resistance and mother-to-child transmission. J Med Virol. 2016; 88(11):1936–43. doi: 10.1002/jmv.24540 27037910 10.1002/jmv.24540

[38] Delwart EL, Shpaer EG, Louwagie J, McCutchan FE, Grez M, Rubsamen-Waigmann H, et al Genetic relationship determined by a DNA heteroduplex mobility assay: analysis of HIV-1 env genes. Science. 1993; 19;262(5137):1257–61. 8235655 10.1126/science.8235655

[39] Sierra M, Thomson MM, Rios M, Casado G, Castro RO, Delgado E, et al The analysis of near full-length genome sequences of human immunodeficiency virus type 1 BF intersubtype recombinant viruses from Chile, Venezuela and Spain reveals their relationship to diverse lineages of recombinant viruses related to CRF12_BF. Infect. Genet. Evol. 2005; 5,209–217. doi: 10.1016/j.meegid.2004.07.010 15737911 10.1016/j.meegid.2004.07.010

[40] Passaes CP, Bello G, Lorete RS, Matos Almeida SE, Junqueira DM, Veloso VG, et al Genetic characterization of HIV-1 BC recombinants and evolutionary history of the CRF31_BC in Southern Brazil. Infect Genet Evol. 2009; 9(4):474–82. doi: 10.1016/j.meegid.2009.01.008 19460312 10.1016/j.meegid.2009.01.008

[41] Thompson JD, Higgins DS, Gibson TJ. CLUSTAL W: improving the sensitivity of progressive multiple sequence alignment through sequence weighting, position-specific gap penalties and weight matrix choice. Nucleic Acids Res. 1994; 22:4673–4680. 7984417 10.1093/nar/22.22.4673 PMC308517

[42] Nei M & Kumar S. Molecular Phylogenetics and Evolution. Oxford University. 2002; 3:567–568

[43] Kimura M. A simple method for estimating evolutionary rates of base substitutions through comparative studies of nucleotide sequences. J Mol Evol. 1980; 16:111–120. 7463489 10.1007/BF01731581

[44] Tamura K, Stecher G, Peterson D, Filipski A, Kumar S. MEGA6: Molecular Evolutionary Genetics Analysis Version 6.0. Mol Biol Evol. 2013; 30(12): 2725–2729. doi: 10.1093/molbev/mst197 24132122 10.1093/molbev/mst197 PMC3840312

[45] Lole KS, Bollinger RC, Paranjape RS, Gadkari D, Kulkarni SS, Novak NG, et al Full-length human immunodeficiency virus type 1 genomes from subtype C-infected seroconverters in India, with evidence of intersubtype recombination. J Virol. 1999; 73(1):152–160. 9847317 10.1128/jvi.73.1.152-160.1999 PMC103818

[46] Xia X., Xie Z. DAMBE: software package for data analysis in molecular biology and evolution. J. Hered. 2001; 92,371–373. 11535656 10.1093/jhered/92.4.371

[47] Drummond AJ, Nicholls GK, Rodrigo AG, Solomon W. Estimating mutation parameters, population history and genealogy simultaneously from temporally spaced sequence data. Genetics. 2002; 161:1307–1320. 12136032 10.1093/genetics/161.3.1307 PMC1462188

[48] Drummond AJ, Rambaut A. BEAST: Bayesian evolutionary analysis by sampling trees. BMC Evol Biol. 2007; 7:214 doi: 10.1186/1471-2148-7-214 17996036 10.1186/1471-2148-7-214 PMC2247476

[49] Suchard MA, Rambaut A. Many-core algorithms for statistical phylogenetics. Bioinformatics. 2009; 25:1370–1376. doi: 10.1093/bioinformatics/btp244 19369496 10.1093/bioinformatics/btp244 PMC2682525

[50] Drummond AJ, Rambaut A, Shapiro B, Pybus OG. Bayesian coalescent inference of past population dynamics from molecular sequences. Mol Biol Evol. 2005; 22:1185–1192. doi: 10.1093/molbev/msi103 15703244 10.1093/molbev/msi103

[51] Drummond AJ, Ho SY, Phillips MJ, Rambaut A. Relaxed phylogenetics and dating with confidence. PLoS Biol. 2006; 4:e88 doi: 10.1371/journal.pbio.0040088 16683862 10.1371/journal.pbio.0040088 PMC1395354

[52] Rambaut A, Drummond A. 2007 Tracer v1.6. Available from http://treebioedacuk/software/tracer/

[53] Carr JK, Avila M, Gomez Carrillo M, Salomon H, Hierholzer J, Watanaveeradej V, et al Diverse BF recombinants have spread widely since the introduction of HIV-1 into South America. AIDS. 2001; 19;15(15):F41–7. 11600844 10.1097/00002030-200110190-00002

[54] Bello G, Aulicino PC, Ruchansky D, Guimarães ML, Lopez-Galindez C, Casado C, et al Phylodynamics of HIV-1 Circulating Recombinant Forms 12_BF and 38_BF in Argentina and Uruguay. Retrovirology. 2010; 7:22 doi: 10.1186/1742-4690-7-22 20307282 10.1186/1742-4690-7-22 PMC2854103

[55] Ristic N, Zukurov J, Alkimim W, Diaz RS, Janini LM, Chin MP. Analysis of the origin and evolutionary history of HIV-1 CRF28_BF and CRF29_BF reveals a decreasing prevalence in the AIDS epidemic of Brazil. PLoS One. 2011; 6(3):e17485 doi: 10.1371/journal.pone.0017485 21390250 10.1371/journal.pone.0017485 PMC3046974

[56] De Sa-Filho DJ, Ambar RF, Duarte NB, Matias RB, Candido V, Gagliani LH, et al HIV type 1 diversity from newly diagnosed patients in Santos metropolitan area/Brazil. AIDS Res Hum Retroviruses. 2009; 25(9):925–9. doi: 10.1089/aid.2009.0073 19689200 10.1089/aid.2009.0073

[57] Monteiro-Cunha JP, Araujo AF, Santos E, Galvao-Castro B, Alcantara LC. Lack of high-level resistance mutations in HIV type 1 BF recombinant strains circulating in northeast Brazil. AIDS Res Hum Retroviruses. 2011; 27(6):623–31. doi: 10.1089/AID.2010.0126 21087197 10.1089/AID.2010.0126

[58] Santos LA, Monteiro-Cunha JP, Araujo AF, Brites C, Galvao-Castro B, Alcantara LC. Detection of distinct human immunodeficiency virus type 1 circulating recombinant forms in northeast Brazil. J Med Virol.2011; 83(12):2066–72. doi: 10.1002/jmv.22170 22012712 10.1002/jmv.22170

[59] Barreto CC, Nishyia A, Araújo LV, Ferreira JE, Busch MP, Sabino EC. Trends in antiretroviral drug resistance and clade distributions among HIV-1 infected blood donors in Sao Paulo, Brazil. J Acquir Immune Defic Syndr. 2006; 41(3):338–41. doi: 10.1097/01.qai.0000199097.88344.50 16540943 10.1097/01.qai.0000199097.88344.50

[60] Prellwitz IM, Alves BM, Ikeda ML, Kuhleis D, Picon PD, Jarczewski CA, et al HIV behind bars: human immunodeficiency virus cluster analysis and drug resistance in a reference correctional unit from southern Brazil. PLoS One.2013; 9;8(7):e69033 doi: 10.1371/journal.pone.0069033 23874857 10.1371/journal.pone.0069033 PMC3706441

