# Supplementary material for: Characterization of HIV-1 CRF90_BF1 and putative novel CRFs_BF1 in Central West, North and Northeast Brazilian regions
Source: PLoS One. 2017 Jun 19;12(6):e0178578. doi: 10.1371/journal.pone.0178578 (PMC5476242; doi:10.1371/journal.pone.0178578)
Supplement: S1 Table — Pregnant: women infected with HIV-1 attending a regional antenatal care; Naïve: antiretroviral naïve patients; HAART: Patients under highly active antiretroviral therapy. * Ref 29: the study group (n = 27) comprises prisoner patients recruited in Goiania/GO (n = 7) and in Campo Grande (n = 20). (DOCX) [file pone.0178578.s001.docx]

**S1 Table. Prevalence of BF1 recombinants identified in previous studies among patients from six Brazilian States: Goiás/ GO, Mato Grosso/ MT, Mato Grosso do Sul/MS, Tocantins/TO, Piauí/PI and Maranhão/MA**

| Reference | Study Population | Sample Collection (Year) | HIV-1 sequences (n) | BF1 frequency n (%) | City/State/Region |
| --- | --- | --- | --- | --- | --- |
| Cardoso et al 2009 ^11^ | Naïve | 2007-2008 | 97 | 07 (7.2) | Goiânia/GO/Central West |
| Cardoso & Stefani 2009 ^27^ | HAART | 2007-2008 | 48 | 07 (14.6) | Goiânia/GO/Central West |
| Cardoso et al 2010 ^28^ | Pregnant | 2003 | 77 | 13 (16.9) | Goiânia/GO/Central West |
| Cardoso et al 2011^29*^ | Prisoner | 2008-2009 | 27 | 01 (3.7) | Goiânia/GO/Central West |
| Cardoso et al 2011^29*^ | Prisoner | 2008-2009 | 27 | 07 (25.9) | Campo Grande/MS/Central West |
| Ferreira et al 2011 ^30^ | Naïve | 2008-2009 | 92 | 11 (11.9) | Cuiabá/MT/Central West |
| Carvalho et al 2011 ^31^ | Naïve | 2008-2009 | 52 | 04 (8.2) | Palmas/TO/North |
| da Silveira et al 2012 ^32^ | Naïve | 2008-2010 | 49 | 04 (7.7) | Campo Grande/MS/Central West |
| Alcântara et al 2012 ^33^ | Pregnant | 2008-2010 | 83 | 15 (18.1) | Goiânia/GO/Central West |
| da Costa et al 2013 ^34^ | Pregnant | 2010-2011 | 18 | 02 (11.1) | Goiânia/GO/Central West |
| Moura et al 2015a ^35^ | Naïve | 2012-2013 | 106 | 08 (7.5) | São Luiz /MA/Northeast |
| Moura et al 2015b ^36^ | Naïve | 2011-2012 | 89 | 04 (4.5) | Teresina /PI/Northeast |
| Lima et al 2016 ^37^ | Pregnant | 2010-2013 | 90 | 06 (6.7) | Goiânia/GO/Central West |

Pregnant: women infected with HIV-1 attending a regional antenatal care; Naïve: antiretroviral naïve patients; HAART: Patients under highly active antiretroviral therapy. * Ref 29: the study group (n=27) comprises prisoner patients recruited in Goiania/GO (n=7) and in Campo Grande (n=20).
