# Supplementary material for: Characterization of HIV-1 CRF90_BF1 and putative novel CRFs_BF1 in Central West, North and Northeast Brazilian regions
Source: PLoS One. 2017 Jun 19;12(6):e0178578. doi: 10.1371/journal.pone.0178578 (PMC5476242; doi:10.1371/journal.pone.0178578)
Supplement: S2 Table — *Some primers had their original sequence modified based on the alignment of subtypes B, C and F1 HIV sequence compendium (2005) from HIV Los Alamos Database. (DOCX) [file pone.0178578.s002.docx]

**S2Table. List of HIV-1 primers used in the present study for full length genome amplification**

| **Name of Primers** | **Fragment #** | **Primer Sequence (5’ 🡪 3’)** |
| --- | --- | --- |
| SCAOSD | 1 | GGGACTTTCCGCTGGGGACTTTC |
| LR51 | 1 | GTATTCCTAATTGAACYTCC |
| SCANSD | 1 | CGAGCCCTCAGATGCTGCATATAAGC |
| DP11 | 1 | CCTGGCTTYAATTTTACTGGTA |
| GAG1AS | 1 | CTTAATAYTGACGCTCTCGC |
| H1G777AS | 1 | AGAACTTTRAAYGCATGG |
| MZ14 | 1 | GAACCKRTCTACATAGTCTC |
| H1P202 | 1 | CTAATACTGTATCATCTGCTCCTG |
| GAG3 | 1 | CTRGGATTAAATAAAATAGT |
| MZ14S | 1 | GAGACTATGTAGAYMGGTTC |
| P24-1 | 1 | CCCTGRCATGCTGTCATCA |
| SCCNAS | 2 | TGTCCCTGTAATAAACCCGAAAATTTTG |
| DP10 | 2 | CAACHCCCTCTCMGAAGCAGGAGCCG |
| SCCOAS | 2 | TATTCTTTCCCCTGCACTGTA |
| DP16 | 2 | CCTCARRTCACTCTTTGGCARC |
| LR49 | 2 | CAATGGCCATTGACAGAAGA |
| LR51 | 2 | GTATTCCTAATTGAACYTCC |
| SEQRT | 2 | GGATGGAAAGGATCACCAGCAA |
| MMRT6 | 2 | TTTTACATCATTAGTGTGGG |
| MMRT9 | 2 | AACAAACTCCCAYTCAGGA |
| MMRT3 | 2 | TGTGCTGGTACCCATG |
| MMINT4 | 2 | CTTGACTTTGGGGATTGTAGGG |
| MMINT8 | 3 | GCAGGAAGATGGCCAGT |
| ED12 | 3 | AGTGCTTCCTGCTGCTCCCAAG |
| ED14 | 3 | TGYCTGGAGCTGYTTRATGCCCCAGAC |
| MMINT3 | 3 | CCCTACAATCCCCAAAGTCAAG |
| MMINT6 | 3 | GGGATGTGTACTTCTGAACTT |
| ED3 | 3 | TTAGGCATYTCCTATGGCAGG |
| ED3AS | 3 | CCTGCCATAGGARATGCCTAA |
| ENV01AS | 3 | CTTTGGACARGCYTGTGTWATGRYTGAGG |
| ENV04AS | 3 | ATTTCTRGGTCCCCTCCTGA |
| ED5 | 4 | ATGGGATCAAAGCCTAAARCCATGTG |
| SCDOAD | 4 | AGTCACACAACAGACGGGCACACAC |
| JH44 | 4 | ACAGTRCARTGYACACATGG |
| LTR2 | 4 | AGGCAAGCTTTATTGAGGCTTAAG |
| ED33S | 4 | GAGGRGAATTTTTCTAYTGYAA |
| MM4 | 4 | CCTCCTACTATCATTATGAA |
| SCDOS | 4 | CCAYTAGGARTAGCACCCAC |
| NEF6AS | 4 | GTCATTGGTCTTAAAGGYAC |
| LTR1 | 4 | CACACACAAGGCTACTTCCCT |
| NEF3 | 4 | GRACAGATAGRRTYATAGAA |
| LTR2 | 4 | AGGCAAGCTTTATTGAGGCTTAAG |

*Some primers had their original sequence modified based on the alignment of subtypes B, C and F1 HIV sequence compendium (2005) from HIV Los Alamos Database.
